# Measuring psychological resilience to disasters: are evidence-based indicators an achievable goal?

**DOI:** 10.1186/1476-069X-12-115

**Published:** 2013-12-20

**Authors:** Jose Manuel Rodriguez-Llanes, Femke Vos, Debarati Guha-Sapir

**Affiliations:** 1 Centre for Research on the Epidemiology of Disasters, Institute of Health and Society, Université catholique de Louvain, Clos Chapelle-aux-Champs 30, Brussels 1200, Belgium

**Keywords:** Psychology, Mental health, Resilience, Recovery, Disaster, Conflict, Risk factor, Indicator, Policy, Review

## Abstract

Despite rising interest on the concept of societal resilience and its measurement, little has been done to provide operational indicators. Importantly, an evidence-based approach to assess the suitability of indicators remains unexplored. Furthermore few approaches that exist do not investigate indicators of psychological resilience, which is emerging as an important component of societal resilience to disasters. Disasters are events which overwhelm local capacities, often producing human losses, injury and damage to the affected communities. As climate hazards and disasters are likely to increase in the coming decades, strengthening the capacity of societies to withstand these shocks and recover quickly is vital. In this review, we search the Web of Knowledge to summarize the evidence on indicators of psychological resilience to disasters and provided a qualitative assessment of six selected studies. We find that an evidence-based approach using features from systematic reviews is useful to compile, select and assess the evidence and elucidate robust indicators. We conclude that strong social support received after a disaster is associated with an increased psychological resilience whereas a female gender is connected with a decrease in the likelihood of a resilient outcome. These results are consistent across disaster settings and cultures and are representative of approximately 13 million disaster-exposed civilians of adult age. An approach such as this that collects and evaluates evidence will allow indicators of resilience to be much more revealing and useful in the future. They will provide a robust basis to prioritize indicators to act upon through intersectoral policies and post-disaster public health interventions.

## Review

### Background

The science of resilience is emerging rapidly, boosted up by increased awareness in the policy circles [1]. The concept of resilience derives from a complex, rich and long history and the term is currently used in many fields where it adopts different meanings [2]. In fields connected with Disaster Risk Reduction, divergent definitions of resilience have also been noted [3]. For clarity purposes, here we adopted the same definition as the Intergovernmental Panel on Climate Change, which describes resilience as “the ability of a system and its component parts to anticipate, absorb, accommodate, or recover from the effects of a hazardous event in a timely and efficient manner, including through ensuring the preservation, restoration, or improvement of its essential basic structures and functions” [4].

Europe will face important environmental and social challenges in the next decades. Recent research predicts important losses in household welfare and health due to climate change in Europe by 2080, in absence of adequate adaptation [5]. As occurred with past economic crises, the current ongoing one has been suggested as already affecting important aspects of human health and well-being [6-8]. The current crisis will probably contribute to set a worse overall health status for the populations which will face new adversities. Neither will the evolving global economic situation will be helped by the already increased number of disasters and associated economic losses [9], which were reported to be especially costly in most recent years [10], and are expected to worsen in a foreseeable future [10,11].

In this context, improving our capacity to adapt effectively in a rapidly changing world will be crucial in the years to come, in the developing as well as in the developed world. The creation of disaster resilient societies, or those able to absorb the impact and bounce back in a timely manner from any disturbance, is seen today as a desirable target to achieve in order to make our societies safer while contributing to their sustainability [12]. But how can the concept of resilience be operationalized to help policy makers in their mission? Measuring resilience is one critical element of the chain; however it is a challenging task, as resilience is a complex construct whose understanding requires multidisciplinary perspective and input. A recurrent problem that has been cited by many authors is the lack of a clear definition of resilience [13,14]. Others have also pointed at the heterogeneity of available research that hinders the overall assessment of findings, for example, through meta-analysis [13].

A second crucial element is the production of indicators. In general, the literature shows that we can differentiate between resilience as an outcome measure and as an indicator. The first concept shows resilience post-facto, i.e. once the disturbance has interacted with a community. Low mortality and low injury rates, absence of or low posttraumatic stress disorder (PTSD) symptoms among the exposed, or high rates of timely relocation of the displaced due to disasters are examples of resilience outcomes. The second concept generally refers to baseline conditions measurable in a community ante-facto. Those attributes, called here resilience indicators, have the potential to predict disaster resilience within that community before a disaster occurs [1].

Resilience indicators that can be measured ante-facto are important to inform policy. These indicators, which can be altered to improve resilience, interestingly target the side of prevention. Likewise, assessed communities might be compared on their levels of resilience, pinpointing those communities with lower resilience levels. Thus their use is important for resource allocation [1]. The efficiency of this approach might be further amplified if the indicators apply to all-hazards versus a single-hazard approach. Hitherto this has been suggested as being difficult in both ecological systems [15] and human societies [16].

The present work is largely motivated by two major gaps in the literature. First of all, little has been done to develop operational indicators of resilience [1,15,16]. Particularly psychological resilience, acknowledged as one of the main constructs of societal resilience, has never been targeted in the development of policy-actionable indicators [1]. Second, a common practice has been to select indicators according to issues of data availability, rather than upon the best available scientific evidence; such constrained approaches are almost inevitably more subject to bias [17]. Despite empirical evidence is growing rapidly within the field, there is a lack of clear methods to assess and select those indicators that likely measure the characteristics of interest. What is sound evidence? How can we compare it? In this sense, systematic reviews are widely and successfully used in the medical sciences to provide an answer to a clearly formulated question and might be of practical use here [18]. Systematic reviews use methodical and unambiguous approaches to locate, select, assess, compile and analyze - quantitatively or qualitatively, empirical evidence [19]. Here we review the scientific literature providing indicators of psychological resilience to disasters using the Web of Knowledge^SM^ (WoK). The aim is to test the usefulness of systematic reviews as a plausible approach to produce evidence-based indicators of resilience to disasters.

## Methods

We examined, by means of the WoK, the available literature on indicators that show psychological resilience to disasters, and in which resilient outcomes are clearly identified. We selected this evidence using predefined key terms and a search strategy, including clear inclusion and exclusion criteria to select any empirical evidence.

### Selection of key terms and search strategy

We first selected the key terms for our search related to indicators of psychological resilience to natural disasters. We based this selection on key scientific articles and previous work of the emBRACE consortium. The list of terms attempted to capture three main components, using main terms and synonyms to identify: a) outcomes (of psychological resilience); b) the event (disasters and/or other stressors); and c) indicators of resilience.

To have an initial estimation of the amount of sensibility and specificity of our search, an initial test search was made in WoK with the key words and synonyms for ‘psychological resilience’, ‘natural disasters’ and ’indicator’ (Additional file 1: Table S1). Next, key words for events other than natural disasters were added, such as ‘traumatic events’ and ‘terrorist attack’, since the number of studies focused on natural disasters was relatively small (Additional file 1: Table S1). Additional key words for factors that are related to psychological resilience, such as ‘coping behaviour’ and ‘positive emotion’, were further investigated (Additional file 1: Table S1). The outcome of each search was stored, and articles were scrutinized to estimate their relevance based on title, abstract and key words. Based on this preliminary overview, final key words for the literature review were selected (“Key terms used and search strategy used in this review” subsection).

### Key terms used and search strategy used in this review

#### Resilient outcome

TS=("psychological resilienc*" OR "psychosocial resilienc*")

#### Disaster/Stressor

AND (disaster* OR hazard* OR catastrophe* OR earthquake* OR volcano* OR "mass movement*" OR storm* OR flood* OR "extreme temperature*" OR drought* OR wildfire* OR "wild fire*" OR rockfall* OR landslide* OR avalanche* OR subsidence OR "storm surge*" OR "heat wave*" OR heatwave* OR "cold wave*" OR coldwave* OR "extreme winter condition*" OR inundation* OR windstorm* OR "industrial accident*" OR "transport accident*" OR "terrorist attack*" OR "potentially traumatic event*" OR “traumatic event*” OR “adverse event*” OR “extreme event*” OR “psychological trauma” OR conflict OR war OR violence OR adversity)

#### Indicator of resilience

AND (factor* OR indicator* OR variable* OR characteristic* OR examination* OR assessment* OR measure* OR association* OR predictor* OR determinant* OR psychometric*)

For truncated search terms (e.g. resilienc*), a search is submitted for all words starting with these letters and would in this example search for “resilience,” “resiliency,” etc. For non-truncated search terms with “*” (e.g. disaster*), a search for plurals of the term (e.g. disasters) is performed.

The literature search was performed in the WoK using the access to the “All Databases” form with key words in the “Topic field” (including searches in Title, Abstract, Author Keywords and Keywords Plus®). The time span was set at “All years”, which includes all published articles from 1969 to the 25^th^ January 2013. The search in WoK resulted in 58 references.

### Exclusion criteria

We excluded articles on non-civilian populations or distant populations nearly unaffected, those articles that did not study indicators of psychological resilience to disasters (e.g., cancer). We were not restrictive in the definition of psychological resilience, but we excluded studies that did not define and measure psychological resilience as the study outcome (e.g. studies measuring the prevalence of posttraumatic stress disorder after disasters were discarded). Finally, book chapters, editorials, as well as studies written in a language other than English were left out of this review (Additional file 2: Table S2).

### Screening on title and abstract

Full references were stored in a Microsoft Office Access database management system. As a first step, the collected references were screened by title to make a preliminary selection. Six articles were excluded as they were considered outside the scope of this review (Additional file 2: Table S2). Second, the remaining 52 references were screened on the abstracts by two researchers independently. Discrepancies between the outcomes of the screenings were jointly discussed and a common final decision on articles to be retained for further review was made. In all, twenty-three articles were excluded based on the screening of abstracts (Additional file 2: Table S2).

### Review of full text articles

Subsequently, the full text of the 29 selected articles was carefully read. Based on the full text review, 13 articles were excluded. A total of 16 articles were used for further investigation, namely to develop an overview table summarizing the available evidence, and to organize and evaluate the relevance and consistency of the available evidence based on further criteria.

From each paper we extracted relevant information on methods, study design, populations targeted, and outcome and predictive variables, as well as measures of effect size with their statistical significance, if available. Only a set of these variables, relevant to the study objectives, were reported here (Table 1).

**Table 1:** Key methodological features of the 6 analyzed studies

**Authors, year**	**Event, location and year**	**Study population (sampling frame)**	**Study design**	**Sample size**	**Analysis**	**Sample representative of population**
Bonanno et al., 2008	SARS epidemic, Hong Kong 2003	Hospitalized SARS adult (≥ 18 years) survivors tracked by the Hong Kong Hospital Authority (N = 1,331). Total of 1,775 individuals infected by SARS in Hong Kong	A face-to-face longitudinal study, including 3 interviews at 6, 12, and 18 months after SARS-related hospitalization	n = 951 (6 months); n = 977 (12 months); n = 856 (18 months)	Latent class growth curve modeling (test the association of a trajectory with a set of predictors)	Approximated well Hong Kong's population characteristics, except by having a higher proportion of women (59.2%) compared to the 2001 census (51.7%). All analysis controlled by this factor
Lee et al., 2009	Hurricane Katrina, New Orleans (USA) 2005	African American Hurricane Katrina evacuees aged 18 or older living in New Orleans area but residing in Houston, Texas, in emergency shelters (N ≈ 8,000 evacuees)	A face-to-face cross-sectional survey, administered on a random sample of evacuees in emergency shelters located in Houston, Texas (Kaiser Washington Post Harvard Poll #2005 WPH020) within one month after the hurricane	n = 621, but analysis conducted only on 363 respondents with complete questionnaires (list wise deletion used)	Logistic regression and LISREL analysis (path diagram and path analysis)	No analyses to account for differences were reported
Johannesson et al. 2011	Tsunami, South East Asia 2005	All Swedish citizens registered at Swedish airports during the first weeks after the disaster and older than 16 (N = 10,501)	A longitudinal mail survey using exhaustive sampling 14 months and 36 months after disaster	n = 4,910 at 14 months (T1); n = 3,457 at 36 months (T2)	Analysis of resilient trajectories related to exposure levels and bereavement status (descriptive). Odds Ratios for the association of mental health and each risk factor in multivariate logistic regression analysis (adjusted by all covariates)	Likely, as no difference detected between respondents and non-respondents
Hobfoll et al., 2009	Terrorist attacks, Israel 2004-2005	All Jews and Arabs, 18 years of age of older, living in Israel (N = 4,503,785 according to 2004 census - total population, excluding an estimated 34% of the population younger than 18). Sampling frame selection based on telephone land lines	A face-to-face longitudinal survey on a random sample, including 2 interviews (August-September 2004, August-October 2005) coincident with the latter period of the Second Intifada	n = 1,613 (August-September 2004); n = 709 (August-October 2005)	Analysis of resilient trajectories associated with a set of risk factors using multivariate logistic regressions (adjusting by all covariates with p < 0.01 in bivariate analyses)	Likely, the sample represented the distribution in the Israeli population on gender, age, place of residence and voting behavior
Bonanno et al., 2007	9/11 terrorist attack, New York 2001	Adult (18 and older) citizens in New York City and contiguous geographic areas in New York State, New Jersey, and Lower Fairfield County in Connecticut (N ≈ 6,080,000, according to census 2000, and excluding 24% of the population younger than 18 years)	Random digit-dial household cross-sectional survey with questionnaires administered face-to-face	n = 2,752 approximately 6 months after September 11, 2001	Multivariate logistic regression. Final model selection taking a hierarchical approach (adjusted by all covariates)	Likely, the sample represented the distribution in this population on gender, age, and race
Hobfoll et al., 2012	Chronic exposure to political violence and social upheaval, Palestinian Authority 2007-2008	All citizens of the Palestinian Authority and East Jerusalem older than 18 years (2010 total population is estimated at N ≈ 4,400,000 by United Nations). Around half should be <18 years old giving a rough final figure of 2,200,000	A longitudinal survey including three waves of interview (September-October 2007, April-May 2008, October-November 2008). A stratified three-stage cluster random sampling strategy was used to select the participants. The questionnaire were administered face-to-face	n = 1,196 (initial sample) and n = 769 (analysed)	Multivariate simultaneous equation models (SEM). This model estimates the complex relationship among variables. This analysis also control for other modeled variables.	Unknown, as the authors did not have data to analyze distribution of non-response and similarly they did not have a detailed census to compare with

Sixteen studies (6 empirical papers, 9 were reviews, one theoretical) were retained and indicators of resilience, along with other descriptive variables were extracted. The six empirical studies provided 53 (non-exclusive) indicators of psychological resilience (Table 2).

**Table 2:** Key empirical studies that identify indicators of psychological resilience

**Authors, year**	**Event, location and year**	**Indicators of resilience**	**Effect of the indicator on resilience**	**Resilient outcome (measurement)**
Bonanno et al., 2008	SARS epidemic, Hong Kong (People’s Republic of China) 2003	Physical functioning 6 months after hospitalization	positive	Psychological functioning (SF-12 - MCS) – Resilience trajectory determined by latent class analysis
		Female gender	negative	
		Social support	positive	
		Event-related worry	negative	
Lee et al., 2009	Hurricane Katrina, New Orleans (USA) 2005	Psychological distress	negative	Perceived sense of recovery (a dichotomous variable)
		Income	positive	
		Human loss	negative	
Johannesson et al. 2011	Tsunami, South East Asia 2005	Intensity of exposure	negative	Resilient trajectory (IES-R ≤ 41.6 in two measurements)
		Loss of relatives	negative	
		Highly exposed	negative	Non-impaired mental health (GHQ-12, with cut-off ≥ 3 indicating impaired mental health)
		Female gender	negative	
		Loss of relatives	negative	
		Older age > 60 years	positive	
		Married	positive	
		Childhood trauma	negative	
		More than 3 traumas in adulthood	negative	
		Recent trauma	negative	
		Previous psychiatric illness	negative	
		Social support	positive	
Hobfoll et al., 2009	Terrorist attacks, Israel 2004-2005	Ethnic majority	positive	Recovery trajectory (here called resilience recovery) Initial symptoms related to traumatic stress (17-item PTSD Symptom Scale) and depressive mood (5-item measure of depressive symptoms from the Patient Health Questionnaire) followed by recovery
		Income	positive	
		Psychosocial resource loss	negative	
		Traumatic growth	negative	
		Male gender	positive	Resilient trajectory (here called resistance) is defined by absence of traumatic (17-item PTSD Symptom Scale) or depression symptoms (5-item measure of depressive symptoms from the Patient Health Questionnaire) at both points in time
		High income	positive	
		Being secular	positive	
		Higher education	positive	
		Ethnic majority	positive	
		Psychosocial resource loss	negative	
		Social support	positive	
Bonanno et al., 2007	9/11 terrorist attack, New York (USA) 2001	Female gender	negative	Having 1 or 0 PTSD symptoms (National Women’s Study PTSD module) at any point in the first 6 months after event
		Age > 65 year	positive	
		Asian race/ethnicity	positive	
		College degree	negative	
		Depression	negative	
		Marihuana use	negative	
		Having an income decline	negative	
		Having 1 or 2 chronic diseases	negative	
		Having 3 or more chronic diseases	negative	
		Having a medium-low level of social support	negative	
		Being directly affected by event	negative	
		Having 1 additional recent life stressor	negative	
		Having 2 or more additional recent life stressors	negative	
		Having 2 or 3 prior traumas	negative	
		Having 4 or more prior traumas	negative	
		Experiencing post-event trauma	negative	
Hobfoll et al., 2012	Chronic exposure to political violence and social upheaval, Palestinian Authority 2007-2008	High social support	positive	Engagement, defined as a persistent, pervasive and positive affective-motivational state of fulfillment (8-item adapted from Schaufeli, Salanova, González-romá and Bakker 2002)
		Resource loss	negative	
		High traumatic exposure	positive	
		Male gender	positive	
		Being more educated	positive	
		Younger	positive	
		Religiosity	positive	

PTSD, post-traumatic stress disorder; IES-R, impact of event scale-revised; SF-12 – MCS, short form 12 (items) – mental component summary; GHQ-12, general health questionnaire 12 (items).

Within the nine reviews and one theoretical study, a total of 135 (non-exclusive) resilience indicators were initially identified. For 36 indicators their effect on resilience was unclear or unreported (Additional file 3: Table S3 and Additional file 4: Table S4). To facilitate visualization, we separated the indicators identified in two reviews that focused exclusively on disasters (Additional file 3: Table S3) from those extracted from reviews focusing more generally on potentially traumatic events (PTEs, Additional file 4: Table S4). PTEs refer also to disasters but may include single traumatic events, such as the loss of friends or relatives, traumatic injury, stress, etc. Because disasters are often multi traumatic events, including many of the previous stressors as well as a life-threatening experiences, resource loss, increased risk of disease and displacement, we prioritized and analyzed here those approaches focused on disasters.

Indicators from empirical studies focusing on other stressors as well as reviews were used as a comparison but not as the main source of analysis in this study. The main reason was that evidence arising from reviews was very heterogeneous: stressors were often not reported, the direction of effect was often missing, as well as a description of the resilient outcomes. Importantly, the review methodology was reported in none of the nine studies. Thus the evidence considered in this study comes from 6 empirical studies which provided 53 indicators. Further, these indicators were grouped in more homogeneous categories and a qualitative evaluation of the evidence was performed based on the consistency of the effect of each indicator on psychological resilience across studies.

It is important to note that the six empirical studies analyzed here were heterogeneous for a number of characteristics, mainly the resilient outcomes, which precluded a meta-analysis on this sample of studies, but also on the disaster types and disaster severity. In detail, these are: a) Resilient outcomes differed in terms of the variables used to measure resilience (e.g., absence of PTSD, low depression, low stress reactions, high level of wellbeing); b) The set of variables (indicators) tested differed across empirical studies; c) Disasters were diverse and with different levels of severity and destruction, from very high, such as the terrorist attacks of September 11 in New York, to lower in the Severe Acute Respiratory Syndrome (SARS) epidemic in Hong Kong.

On the other side, all studies were technically well conducted, used state of the art statistical analysis and considered large cohorts. More in detail: a) All studies focused on adult populations, 18 or older age, except for the study on Swedish tsunami survivors, for which individuals older than 16 years old were also interviewed; b) The size of the study populations ranged from 1,331 (hospital-based study on SARS epidemics) to around 6 million in the case of New York citizens potentially affected by the 11/9 terrorists attacks. All studies worked on large cohorts, with results potentially generalizable to around 13 million adults exposed to diverse disaster settings and intensities of exposure. Excluding two studies which did not test the plausibility of these generalizations, the results would still apply to 10 million individuals; c) Four studies were longitudinal and two were cross-sectional but all focused on psychological resilience within a period of 3 years after the disaster; d) All studies used multivariate analysis to produce their final results. Each resilient indicator was derived from controlled analysis in which the confounding or moderating effect of other covariates was also considered.

## Results

### Indicators of psychological resilience

Fifty three indicators of psychological resilience were obtained from the six empirical studies that focus exclusively on disaster settings (Table 2). The most consistent indicators of psychological resilience were social support and gender. Five studies in which the association was tested found high levels of social support to be a significant predictor of psychological resilience (Table 3). In the case of gender, this was supported by all except one study which pointed to the same direction of the effect but was not significant. Whereas high levels of social support from relatives and friends increased all studied resilient outcomes, women were found at higher risk of suffering a worse psychological resilient outcome after a disaster.

**Table 3:** Assessment of psychological resilience indicators to disasters based on evidence across studies

**Indicator**	**No. studies with indicator tested**	**No. positive effect on PR**	**No. negative effect on PR**
Social support (high)	5	5	0
Female gender	6	0	5
Exposure level (low)	5	4	0
Previous traumatic experiences	3	0	2
Resource loss (economic or psychosocial)	3	0	3
Human loss (friends or relatives)	2	0	2
Physical and mental health (poor)	4	0	4
**Potential indicators, but with limited or contentious evidence**			
Substance abuse (marijuana)	1	0	1
Event-related worry	1	0	1
Education (high)	5	2	1
Income (high)	5	2	0
Marital status (married or partner)	5	1	0
Older age (>60-65)	6	2	1
Being religious	2	1	1
Ethnicity (minority)	2	1	2

PR, psychological resilience; Positive and negative effects reported here were all statistically significant (p < 0.05).

### Probable indicators of psychological resilience

Previous trauma was assessed as a predictor of psychological resilience in half of the studies [20-22]. Two of these studies provided similar results, with trauma in the past negatively affecting future psychological resilience to disasters. Evidence supporting disaster-exposure level as an indicator of resilience was rather solid too. Four of the five studies found low disaster-exposure levels to predict higher psychological resilience. The loss of relatives or friends was an important predictor of lower psychological resilience in the two studies that clearly tested this hypothesis [22,23]. In all three studies in which resource loss (psychological or economic) was evaluated did the results show a positive association with the outcomes. Whenever tested, comorbid physical and mental health was an important predictor of psychological resilience (Table 2 and Table 3).

### Potential indicators of psychological resilience that shows contrasting results

In general, higher level of education was predictive of a resilient psychological outcome. In two of the five studies in which education level was tested the association proved positive. In two other studies no association was found and in the final study a negative association was noted (Table 2 and Table 3). A higher income was connected to a resilient outcome in two studies. No clear effect was found in the other three studies. Marital status -having a partner or spouse- showed limited value as a predictor of a resilient psychological outcome. Despite five studies reported to include this variable, only one found a significant and positive effect on resilience [22]. The effect of age on psychological resilience remained unclear. Although all studies tested the effect of age, only in four studies was this association statistically significant, and the effect was contentious with two studies showing a positive effect of older age on resilience, and another showing a negative effect of older age on resilience.

Hobfoll and collaborators considered religiosity as one important variable in two studies conducted in Israel and the Palestinian Authority respectively (Table 1, Table 2). They found contrasting results with religiosity playing a positive role among Palestinians but having a negative effect on psychological resilience among Israelis [24,25].

Ethnicity played a different role in different settings and its use as an indicator is neither clear nor straightforward. In Bonanno’s study [20], being Asian played a positive role on resilience, while other minorities (other than African American and Hispanic) were at higher risk of a worse psychological outcome. In Hobfoll’s study, being Jewish was associated with higher likelihood of resilience [25].

### Potential indicators of psychological resilience shown by only one study

The association of substance use and resilience was tested in one study. Marijuana use was connected with a decrease in psychological resilience [20] (Table 2). In the same study, alcohol consumption and cigarette use were also tested, but no effect on psychological resilience was found. Presence of event-related worry (fear) in the case of the SARS epidemic was also found to decrease psychological resilience [21].

## Discussion

In this study we identified some of the barriers that need to be considered and solved in the early production of indicators of psychological resilience to disasters. Probably, many of these constraints apply to other areas of resilience. We conducted a review searching the entire WoK to firstly identify evidence-based consistent indicators of psychological resilience to disasters, and secondly to provide a clear methodological approach that might serve as a basis for selection of indicators in future work.

Despite the fact that the evidence on indicators of psychological resilience presents important heterogeneity, a systematic methodology to identify, select, and assess this evidence seems an attainable objective that is confirmed by this work, at least in the case of psychological resilience. The use of this methodology helped us to identify the most robust indicators - social support, female gender, and further probable indicators, i.e. previous trauma, degree of disaster-exposure, human losses, resource loss, and physical and mental health, as the most important ones revealed by our analysis. While many studies reviewed the factors predicting psychological resilience in face of adversities and disasters [13,14,26-31], none of these assessed systematically the literature to provide a list of evidence-based indicators consistent across large civilian populations, different disaster settings and cultural contexts.

Although an emerging gender effect was noticed in some of the articles analyzed by this review, little has been proposed as a potential explanation [20,21,24]. Other authors researching on PTSD had previously suggested different levels of symptom reporting (women reporting more often, men being more reserved) as a plausible reason to explain these differences [32]. As similar methods are used in both areas of research, this might be a plausible explanation for these results. Unfortunately, this work cannot offer further explanations to this question but it is additional evidence supporting the need for focused studies to better comprehend the gender dimension in disasters.

Strikingly, social support, gender, previous trauma, and severity of exposure, were also found as important risk factors for PTSD in trauma-exposed adults [32]. In a more recent review, centered in PTSD after disasters, some of the most consistent correlates of PTSD, as interpreted by the authors, were female gender, prior traumas, lack of social support, degree of exposure to disasters, as well as comorbid psychiatric conditions [33]. The prominent commonalities observed between PTSD and psychological resilience in disaster context offers a great opportunity for policy makers to act upon those indicators, with a potential to improve both conditions simultaneously.

There are a number of limitations that need consideration. First of all, the results of this work need to be regarded with care, as we did not systematically analyze all sources of scientific evidence that might provide indication of what predicts psychological resilience to disasters. Once a methodology is discussed and accepted, it should be much easier to upscale the process. Our objective here was not to systematically review all the literature but rather to propose a methodology tested in a sample. Second, the studies only consider observable variables (indicators) that were considered by the researchers conducting each study. In addition, variables might differ in the way they were studied or coded for analysis. We reported the number of studies in which similar predictors were used as a way as to estimate their presence across the studies and also to estimate the proportion of significant – positive or negative – effects of each variable on psychological resilience. Third, the heterogeneity found in resilient outcomes and study designs precluded meta-analysis. As an alternative, we used a different approach considering as plausible indicators only those in which a majority of studies pointed to the same direction of the effect. Fourth, our approach did not capture a number of indicators focused on personality traits (e.g. positiveness, hardiness) that enhance resilience and that were noted in some of the analyzed reviews [26]. These will be likely captured by future systematic reviews.

Regarding the methods, a number of barriers that deserve further discussion were observed during this work and are commented in the following section. The scientific literature is populated with studies about psychological resilience after normal life-course traumatic experiences [34]. Disasters, in our opinion, might be seen as distinct events in the sense that they often produce many losses among the exposed and many people are simultaneously affected. In the worst situations, survivors might have lost relatives, friends, and dwellings. They themselves might have been injured during the event or have suffered a life-threating experience, might be at higher risk of contracting a number of diseases, be more disabled than before the disaster, and their economic capacity might shrink with rising unemployment rates and the rise of prices that often occur after some disasters. We thus think that disasters are special events that challenge human and community capacities. As such, in this study, we made a first distinction between studies focusing on disasters and non-disaster events.

We were initially interested in a comparison of indicators arising from both studies on disasters and further stressors. If the relevant underlying mechanisms that increase psychological resilience are similarly identified in disasters and non-disasters settings, this evidence might be used as additional criteria to select indicators. However, of the articles examined, only three were relevant but presented some limitations that excluded further comparison. Two of them assessed resilience based on the 10 and 17-item Connor-Davidson Resilience Scale respectively [35,36], which is rather a measure of future individual’s resilience than an outcome. One of these studies focused also on intensive care unit nurses, who cannot be assumed to be representative of the general population [36], the focus of our study. A further report investigated predictors of resilience in trauma injury patients treated in a single facility [37]. Nevertheless, the increasing body of evidence on the factors promoting psychological resilience to stressful life-course events should be seen as an opportunity to further design reviews allowing comparison with indicators of psychological resilience in a disaster setting. As in the case of PTSD, a list of those population conditions promoting benefits on multiple health outcomes (i.e. lowering PTSD, increasing resilience to stress and in disaster-exposed populations) could indeed improve effective policy.

This work, as previous research did [13], also showed that studies on resilience indicators tend to be highly heterogeneous. One option to reduce at least part of this heterogeneity would consist in producing additional exclusion criteria, for example excluding studies that focus on specific population groups, not representative of civilian populations. In this review the analyzed studies represented the general civilian populations older than 18 years and affected by tsunamis, hurricanes, epidemic, conflict and terrorism in Sweden, USA (New York and New Orleans), People's Republic of China (Hong Kong), Israel and the Palestinian Authority. The exception was the study on the Hurricane Katrina, in which only African Americans were represented [23].

For the future development of resilience indicators to disasters, the scientific community might benefit from the use of systematic reviews. Such reviews attempt to answer a specific question and should include: 1) clear inclusion/exclusion criteria to select the available evidence, 2) an explicit search strategy, 3) systematic coding and analysis of included studies, 4) meta-analysis (if possible). We undertook this approach within this work [18]. We think this methodology might be especially useful for the development of indicators, given the apparent high heterogeneity of studies on resilience.

Future studies should expand the number of search engines used, including also the grey literature, to provide a full systematic assessment. Once this objective is accomplished, further issues such as indicator classification, temporal linkages within the disaster cycle, or more technical challenges such as data availability, indicator weighting or scale issues will follow [1,16,38]. The benefits of the above approach may be significant. As resources are never unlimited, evidence-based indicators should be preferable by policy makers to establish a list of priorities for data collection or targeted groups for intervention before and after disasters (e.g., women after this study). Measuring indicators that are evidence-based should be a much more valid approach than following a strategy simply based on data availability, as the likelihood of measuring some relevant components of resilience might increase in the first case compared to a random set of available measures in the second case.

## Conclusions

An evidence-based approach might be helpful to elucidate and prioritize robust indicators of psychological resilience to disasters. Similar methods might be used in other areas of resilience.

Lack of social support, female gender, followed by high exposure level, prior traumas, resource loss, human loss and poor physical or mental health seem to be likely indicators of psychological resilience to disasters. Policies improving these conditions or targeting the most vulnerable groups might be effective in increasing the psychological resilience to disasters.

## Abbreviations

PTSD: Posttraumatic stress disorder; WoK: Web of Knowledge; PTE: Potentially traumatic events; SARS: Severe acute respiratory syndrome.

## Competing interests

The authors declare that they have no competing interests.

## Authors’ contributions

JMR conceived the study, designed the experiments, supervised and contributed to data collection, analyzed and interpreted the data, and wrote the paper. FV collected the data, contributed to study design and data analysis, participated in drafting the methods section of the manuscript, and critically reviewed the paper. DG contributed to study design, helped in drafting the discussion, and critically reviewed the paper. All authors read and approved the final version of this manuscript.

## Supplementary Material

Additional file 1: Table S1 Potentially relevant key words pre-tested in this study. Click here for file

Additional file 2: Table S2 Main reasons for exclusion of published documents during title and abstract screening, and full text review. Click here for file

Additional file 3: Table S3 Review studies that identify potential indicators of psychological resilience to disasters. Click here for file

Additional file 4: Table S4 Review studies that identify potential indicators of psychological resilience to potential traumatic events (PTEs). Click here for file
