# Supplementary material for: Measuring psychological resilience to disasters: are evidence-based indicators an achievable goal?
Source: Environ Health. 2013 Dec 20;12:115. doi: 10.1186/1476-069X-12-115 (PMC3893382; doi:10.1186/1476-069X-12-115)
Supplement: Additional file 1: Table S1 — Potentially relevant key words pre-tested in this study. [file 1476-069X-12-115-S1.doc]

Additional file 1

Table S1. Potentially relevant key words pre-tested in this study.

| **Search 1** | | | **Search 2** | **Search 3** |
| --- | --- | --- | --- | --- |
| **Outcome** | **Event** | **Indicator** | **Event 2** | **Indicators of resilience** |
| psychological resilience | disaster* | factor* | traumatic event* | coping behaviour |
| psychological resiliency | hazard* | indicator* | industrial accident* | psychological adaptation |
| psychosocial resilience | catastrophe* | variable* | transport accident* | psychological response |
| psychosocial resiliency | earthquake* | characteristic* | adverse event* | psychological resources |
|  | volcano* | examination* | terrorist attack* | psychological adjustment |
|  | mass movement* | assessment* | extreme event* | psychological well being |
|  | storm* | measure* | psychological trauma | mental health |
|  | flood* | association* | conflict | demograph* |
|  | extreme temperature* | predictor* | war | exposure |
|  | drought* | determinant* | violence | personality |
|  | wildfire* | psychometric* | adversity | social resources |
|  | wild fire* |  |  | economic resources |
|  | rockfall* |  |  | stressor* |
|  | landslide* |  |  | positive emotion* |
|  | avalanche* |  |  | coping and appraisal |
|  | subsidence |  |  | flexibility |
|  | storm surge* |  |  | individual socio-demography |
|  | heat wave* |  |  | individual resources |
|  | heatwave* |  |  | community resources |
|  | cold wave* |  |  | preparedness and mitigation |
|  | coldwave* |  |  | social support |
|  | extreme winter condition* |  |  | spirituality |
|  | inundation* |  |  | disaster impact severity |
|  | windstorm* |  |  | disaster experience* |
|  |  |  |  | positive adjustment |
|  |  |  |  | positive emotion* |
|  |  |  |  | adaptive capacity |
|  |  |  |  | vulnerability |
|  |  |  |  | PTSD |
|  |  |  |  | psychological recovery |

Search based on key terms identified in reviews (includes emBRACE project reports), key papers, and the authors’ experience.
