# Supplementary material for: Measuring psychological resilience to disasters: are evidence-based indicators an achievable goal?
Source: Environ Health. 2013 Dec 20;12:115. doi: 10.1186/1476-069X-12-115 (PMC3893382; doi:10.1186/1476-069X-12-115)
Supplement: Additional file 2: Table S2 — Main reasons for exclusion of published documents during title and abstract screening, and full text review. [file 1476-069X-12-115-S2.doc]

Additional file 2

**Table S2.** Main reasons for exclusion of published documents during title and abstract screening, and full text review.

| **Main reasons for exclusion** | **No. of articles** |
| --- | --- |
| **Title screening** (6 articles excluded) |  |
| Decisional conflict of cancer patients when choosing treatment | 1 |
| Response of stock market to long periods of terrorism | 1 |
| Psychological resilience in adults undergoing genetic testing for cancer | 1 |
| Editorial comment on study of psychological resilience in veterans | 1 |
| Language other than English | 1 |
| Academic performance in children with divorced parents | 1 |
| **Abstract screening** (23 articles excluded) |  |
| Prevalence of psychopathology | 3 |
| No resilience indicators studied | 6 |
| Non civilian populations (study on veterans) | 6 |
| Long-term resilience / developmental study | 6 |
| Book chapter | 2 |
| **Full text reading** (13 articles excluded) |  |
| Non civilian populations | 2 |
| Cross-sectional data with outcome psychopathology | 2 |
| No indicators of resilience studied | 3 |
| No stressor, confusion of outcome and indicator | 1 |
| Study not representative of the exposed population | 2 |
| Study of unaffected populations | 2 |
| Resilience to traumatic injuries in non-disaster settings | 1 |
