# Supplementary material for: Measuring psychological resilience to disasters: are evidence-based indicators an achievable goal?
Source: Environ Health. 2013 Dec 20;12:115. doi: 10.1186/1476-069X-12-115 (PMC3893382; doi:10.1186/1476-069X-12-115)
Supplement: Additional file 3: Table S3 — Review studies that identify potential indicators of psychological resilience to disasters. [file 1476-069X-12-115-S3.doc]

Additional file 3

**Table S3.** Review studies that identify potential indicators of psychological resilience to disasters.

| Authors,year | Type of event | Indicators of resilience | Effect of the indicator on resilience | Resilient outcome |
| --- | --- | --- | --- | --- |
| Neria et al., 2011[1] | Terrorism, New York 9/11 | Attachment style | not specified | Effective coping during exposure to trauma and reduced psychopathology in its aftermath. |
|  |  | Hardiness | positive |  |
|  |  | Cognitive attributional style | not specified |  |
|  |  | Biological factors | not specified |  |
| Drury and Williams, 2012[2] | War, collective violence, conflict, terrorism | Community acceptance | positive | Protection against distress and/or mental disorders; psychosocial and mental health. |
|  |  | Emotion regulation | positive |  |
|  |  | Parental support | positive |  |
|  |  | Community relations | positive |  |
|  |  | Socio-economic status | positive |  |
|  |  | Family cohesion | positive |  |
|  |  | Perceived support from friends | positive |  |
|  |  | Schools | positive |  |
|  |  | Perceived spiritual support | positive |  |
|  |  | Self-regulation capabilities | positive |  |

**References**

1. Neria Y, DiGrande L, Adams BG: **Posttraumatic stress disorder following the September 11, 2001, terrorist attacks: a review of the literature among highly exposed populations.** *Am Psychol* 2011, **66**:429–446.

2. Drury J, Williams R: **Children and young people who are refugees, internally displaced persons or survivors or perpetrators of war, mass violence and terrorism.** *Curr Opin Psychiatry* 2012, **25**:277–284.
