# Supplementary material for: Measuring psychological resilience to disasters: are evidence-based indicators an achievable goal?
Source: Environ Health. 2013 Dec 20;12:115. doi: 10.1186/1476-069X-12-115 (PMC3893382; doi:10.1186/1476-069X-12-115)
Supplement: Additional file 4: Table S4 — Review studies that identify potential indicators of psychological resilience to potential traumatic events (PTEs). [file 1476-069X-12-115-S4.doc]

Additional file 4

**Table S4. Review studies that identify potential indicators of psychological resilience to potential traumatic events (PTEs).**

| **Authors, year** | **Indicators of resilience** | **Effect of the indicator on resilience** | **Resilient outcome** |
| --- | --- | --- | --- |
| Davydov et al., 2010[1] | Effective coping | positive | A biopsychosocial model of resilience assumes the existence of multiple processes within and outside an organism protecting against disturbance in a manner similar to the complex model of somatic health protection system. |
|  | Positive emotions | positive |  |
|  | Flexible use of emotional resources | positive |  |
|  | Adaptive capacities | positive |  |
|  | Old age | negative |  |
|  | Perceived severity of daily stressors | not specified |  |
|  | Quality of received interpersonal relationships | positive |  |
|  | Quality of perceived social support | positive |  |
|  | Quality of general national, economical, and cultural barriers | positive |  |
|  | Quality of targeted assistance acquired from society | positive |  |
|  | Phenotype advantages | positive |  |
|  | Imprinting, implicit learning | positive |  |
|  | Epigenetic and meaning change mechanisms related to real life adversities (principal in childhood) | positive |  |
|  | ‘Fortification’ programs for general regulation of behaviour or organism functioning in a community | positive |  |
|  | Externally imposed regulation of emotions and behaviours in a person | positive |  |
|  | Cognitive reappraisals related to cognitive therapy | positive |  |
|  | Resilience-promoting interventions | positive |  |
|  | Cognitive appraisal of an event and the emotions induced by the event | not specified |  |
|  | Learning from past events | positive |  |
|  | 5-hydroxytryptamine transporter-linked promoter region (5-HTTLPR) s allele | positive or negative |  |
|  | Aspects of intellectual functioning and cognitive flexibility (positive explanatory style, reappraisal and acceptance) | not specified |  |
|  | Social attachment and social behaviours such as altruism | not specified |  |
|  | Positive selfconcept and effective self-regulation of emotions | not specified |  |
|  | Positive emotions including optimism and humour | not specified |  |
|  | Capacity to convert traumatic helplessness into learned helpfulness | not specified |  |
|  | Meaning including religion/spirituality | not specified |  |
|  | Social support including role models | not specified |  |
|  | Active coping style in confronting a stressor including exercise and training | not specified |  |
|  | Capacity to recover from negative events and stress inoculation | not specified |  |
|  | Capacity to accommodate the new trauma-related information in a positive direction | not specified |  |
|  | Hardiness | not specified |  |
|  | Perceived stress | not specified |  |
|  | Optimism and life attitude | not specified |  |
|  | Adaptive reactivity | not specified |  |
| Bonanno et al., 2011[2] | Personality traits | not specified | A stable trajectory of healthy adjustment across time. |
|  | Perceived control | positive |  |
|  | Trait resilience | positive |  |
|  | negative affectivity | negative |  |
|  | ruminative response style | negative |  |
|  | trait self-enhancement | positive |  |
|  | high perceived coping self-efficacy | positive |  |
|  | Male gender | positive |  |
|  | Older age | positive |  |
|  | Greater education | positive |  |
|  | Exposure | not specified |  |
|  | Emotional support | positive |  |
|  | Social support | positive |  |
|  | Instrumental support (assistance with the tasks of daily living) | positive |  |
|  | Availability of economic resources | positive |  |
|  | Loss of economic resources | negative |  |
|  | Past and current life stress | not specified |  |
|  | Meaning making | not specified |  |
|  | Positive emotions | positive |  |
|  | A priori beliefs (pre-exisiting worldviews) | not specified |  |
| de Terte et al., 2009‡[3] | Cognitions | not specified | Ability of an individual to maintain healthy psychological and physical wellbeing despite being exposed to adversity, including the wider community aspects. |
|  | Emotions | not specified |  |
|  | Behaviours | not specified |  |
|  | Physical activities | not specified |  |
|  | Family support | positive |  |
|  | Community support | positive |  |
|  | Societal support | positive |  |
| Yehuda et al., 2006[4] | Positive affectivity | positive | Ability to bounce back from negative experience, or even significant adversity, by flexible adaptation to the ever-changing demands of life. |
|  | Optimism | positive |  |
|  | Cognitive flexibility | positive |  |
|  | Active coping strategies | positive |  |
|  | Religion/spirituality | positive |  |
|  | Social support and intimacy | positive |  |
|  | Emotion regulation | positive |  |
|  | Mastery | positive |  |
| Cabanyes Truffino, 2010[5] | Control over the process of remembering traumatic experiences | positive | Despite suffering significant traumatic conditions of extreme deprivation, serious threat and major stress, some people manage to endure and recover fully. This unique ability has been called “resilience”. |
|  | Integration of memory and emotions | positive |  |
|  | Regulation of emotions related to trauma | positive |  |
|  | Control of symptoms | positive |  |
|  | Self-esteem | positive |  |
|  | Internal cohesion (thoughts, emotions and actions) | positive |  |
|  | Establishment of secure links | positive |  |
|  | Understanding the impact of the trauma | positive |  |
|  | Developing a positive meaning | positive |  |
|  | Balanced view of one’s life | positive |  |
|  | Perseverance | positive |  |
|  | Self-confidence | positive |  |
|  | Personal autonomy | positive |  |
|  | Meaning of one’s life | positive |  |
|  | Self-efficacy | positive |  |
|  | Self-esteem | positive |  |
|  | Problem-solving | positive |  |
|  | Positive self-concept | positive |  |
|  | Internal locus of control (self-control and emotion regulation) | positive |  |
|  | Personal autonomy | positive |  |
|  | Sense of humour | positive |  |
|  | Social competence | positive |  |
|  | Communication | not specified |  |
|  | Sense of belonging | positive |  |
|  | Empathy | positive |  |
|  | Optimism | positive |  |
|  | Transcendent meaning of life | positive |  |
|  | Religion | positive |  |
| Thompson et al., 2012[6] | Experiential avoidance | negative | Recovery from PTSD or never diagnosed PTSD |
|  | Acceptance | negative | Posttraumatic growth |
| Bonanno and Mancini, 2008[7] | Temperament | not specified | The ability of adults, and children, in otherwise normal circumstances who are exposed to an isolated and potentially highly disruptive event such as the death of a close relation or a violent or life-threatening situation to maintain relatively stable, healthy levels of psychological and physical functioning, as well as the capacity for generative experiences and positive emotions. |
|  | Supportive relations | positive |  |
|  | Community resources | not specified |  |
|  | Pragmatic coping | positive |  |
|  | Adaptive flexibility | positive |  |
|  | Self-enhancement | positive |  |
|  | Repressive coping | positive |  |
|  | Type of exposure | not specified |  |
|  | Duration of exposure | not specified |  |
|  | Intensity of exposure | not specified |  |
|  | Male gender | positive |  |
|  | Age | positive |  |
|  | Education level | positive |  |
|  | Personal and social resources | not specified |  |
|  | Change in resources | negative |  |
|  | Employment loss | negative |  |
|  | Social support loss | negative |  |
|  | Current life stressors | negative |  |
|  | Previous life stressors | negative |  |
| Bonanno and Mancini, 2012[8] | Flexibility in appraisal of PTE | positive | Transient stress reaction that will be mild to moderate in degree and will not significantly interfere with their ability to continue functioning. |
|  | Flexibility in response to PTE | positive |  |
|  | Rigid or context insensitive emotion and coping | negative |  |

‡Theoretical article

**References**

1. Davydov DM, Stewart R, Ritchie K, Chaudieu I: **Resilience and mental health**. *Clin Psychol Rev* 2010, **30**:479–495.
2. Bonanno GA, Westphal M, Mancini AD: **Resilience to loss and potential trauma**. *Annu Rev Clin Psychol* 2011, **7:**511–535.
3. De Terte I, Becker J, Stephens C: **An integrated model for understanding and developing resilience in the face of adverse events**. *J Pac Rim Psychol* 2012, **3**: 20–26.
4. Yehuda R, Flory JD, Southwick S, Charney DS: **Developing an agenda for translational studies of resilience and vulnerability following trauma exposure**. *Ann NY Acad Sci* 2006, **1071:**379–396.
5. Cabanyes Truffino J: **Resilience: an approach to the concept**. *Rev Psiquiatr Salud Ment* 2010, **3**: 145–151.
6. Thompson RW, Arnkoff DB, Glass CR: **Conceptualizing mindfulness and acceptance as components of psychological resilience to trauma**. *Trauma Violence Abuse* 2011, **12:**220–235.
7. Bonanno GA, Mancini AD: **The human capacity to thrive in the face of potential trauma**. *Pediatrics* 2008, **121:**369–375.
8. Bonanno GA, Mancini AD: **Beyond resilience and PTSD: mapping the heterogeneity of responses to potential trauma**. *Psychol Trauma* 2012, **4**:74–83.
